# Effect of Total Dissolved Gas Supersaturation on the Survival of Bighead Carp (*Hypophthalmichthys Nobilis*)

**DOI:** 10.3390/ani10010166

**Published:** 2020-01-18

**Authors:** Yuxuan Deng, Chengyang Cao, Xiaoqing Liu, Quan Yuan, Cuixia Feng, Haoran Shi, Yao Yang, Yufeng Wu

**Affiliations:** 1 Key Laboratory of Fluid and Power Machinery, Ministry of Education, Xihua University, Chengdu 610039, China; dyxstormi@163.com (Y.D.); ccy269147022@163.com (C.C.); fcxgk123@163.com (C.F.); haoranshi21@163.com (H.S.); yy2403265@163.com (Y.Y.); lina199512@163.com (Y.W.); 2 School of Energy and Power Engineering, Xihua University, Chengdu 610039, China; 3 State Key Laboratory of Hydraulics and Mountain River Engineering, Sichuan University, Chengdu 610065, China

**Keywords:** total dissolved gas supersaturation, Bighead carp (*Hypophthalmichthys nobilis*), acute exposure, survival probability, Median survival time (ST_50_)

## Abstract

**Simple Summary:**

Total dissolved gas (TDG) supersaturation severely threatens the survival of fish downstream of hydraulic structures in the Yangtze River due to flood discharge. However, existing findings mostly demonstrate the effect of TDG on the tolerance of benthic fish in China. Few studies have attached great importance to investigating the impact of TDG supersaturation on pelagic fish. Furthermore, varied flow of flood can lead to varied TDG levels in the spill season. Fish resided in the downstream of high dams may experience a period of chronic exposure at a low TDG level before the arrival of flood peak. However, rare achievement has reported that the survival situation of fish exposed to TDG supersaturated water with a high level after receiving chronic exposure. This study investigates the tolerance of bighead carp (*Hypophthalmichthys nobilis*) (pelagic fish) to TDG supersaturation and determine the effect of a high level of TDG supersaturation on its survival after chronic TDG exposure. The results showed that TDG led to apparent abnormal behaviours and gas bubble disease signs in bighead carp. The tolerance of bighead carp declined with increasing TDG levels. Compared with the large juvenile bighead carp, the small juvenile bighead carp are more sensitive to TDG. Furthermore, the results indicated that bighead carp subjected to acute exposure after long term chronic exposure are more vulnerable to TDG. The data from this study may provide valuable information related to protecting aquatic organism diversity and establishing water quality standards related to TDG supersaturation.

**Abstract:**

To assess the effect of TDG on the survival of different sizes of pelagic fish, bighead carp (*Hypophthalmichthys nobilis*) were subjected to TDG supersaturated water at levels of 125, 130, 135, and 140%. The results showed that apparent abnormal behaviours and symptoms of gas bubble disease (GBD) were observed in bighead carp. The survival probability of large and small juvenile bighead carp declined with increasing TDG levels. The median survival time (ST_50_) values of large juvenile bighead carp were 74.97 and 31.90 h at 130% and 140% TDG, respectively. While the ST_50_ of small fish were 22.40 and 6.72 h at the same TDG levels. In comparison to the large juvenile bighead carp, the small juvenile bighead carp showed weaker tolerance to TDG supersaturated water. Furthermore, acute lethality experiments after chronic exposure to TDG were initiated to further investigate the effect of TDG on bighead carp. The juveniles were first subjected to 115% TDG supersaturated water for 96 h. After chronic exposure, live fish were immediately transferred to TDG supersaturated water at levels of 125, 130, 135, and 140%. The results demonstrated that no fish died under chronic exposure and few fish exhibited slight GBD symptoms. The ST_50_ values for bighead carp subjected to acute exposure after chronic exposure were 61.23 and 23.50 h at 130 and 140%, respectively. Compared with the bighead carp subjected to acute exposure, bighead carp subjected to multiple exposures were more vulnerable to TDG.

## 1. Introduction

Flood discharge from high dams can lead to the generation of total dissolved gas (TDG) supersaturation downstream of these dams. Recently, with increasing demand for energy, many dams with high heads have been constructed or have already been completed in China [1,2,3]. In addition, high-level TDG supersaturated water exists downstream of these high dams in the spill season [4,5]. The survival of aquatic organisms (especially fish) may be severely impacted by TDG supersaturation. Previous studies have shown that gas bubble disease (GBD) induced by TDG plays a role in decreasing the survival of fish inhabiting the lower reaches of dams [6]. TDG supersaturation occurred downstream of some hydropower structures in the Columbia River basin due to flood discharge in the spill season and caused the death of chum salmon [7,8,9]. Similar circumstances were also found downstream of the Cabinet Gorge Dam in the Clark Fork River [10]. Moreover, chronic exposure to low TDG levels (less than 120%) might not induce the mortality of fish directly but increase the probability of bacterial and fungal infections in juvenile salmon [11,12,13]. Cramer (1996) indicated that a high TDG level (130%) impacted the survival of chinook smolt downstream of the Ice Harbour Dam (Snake River) after receiving 110–115% TDG chronic exposure upstream of the dam [14]. Moreover, the survival probabilities of in-river-migrating (IR) smolt subjected to multiple exposures of TDG supersaturated water (acute TDG exposure with an average level of 132% after chronic TDG exposure with an average level of 118%) decreased compared to those of smoults subjected to single exposure (only experience lower TDG exposure with an average level of 118%) [15]. Furthermore, Meekin and Turner (1974) indicated that size greatly influenced the tolerance of fish to TDG supersaturation. The mortality of large chinook salmon (100 mm) was three to four times higher than that of small chinook salmon (under 40 mm) [16].

In China, some endemic fish that inhabit the upper Yangtze River were included in the experiments to investigate the effect of TDG supersaturation on the hatching, growth, tolerance threshold and swimming ability of fish [17,18,19,20,21]. Wang et al., (2018) [21] noted that the swimming ability of Prenant’s schizothoracin and Chinese sucker significantly declined with elevated levels of TDG supersaturation. Liang et al., (2013) [17] noted that the median hatching time of David’s schizothoracin increased in conjunction with elevated TDG levels. Decreased hatching rates and increased lesion rates occurred in the high TDG saturation groups. The authors believed that the early life of the species was impacted by TDG supersaturation.

However, these studies have focused on the impact of TDG on the survival of aquatic organisms, mainly benthic fish, and few studies have evaluated the survival of pelagic fish. Furthermore, varied flood discharge flows can lead to varied TDG levels in the spill season. Aquatic organism residing downstream of high dams might experience a period of chronic exposure with a low TDG level before the flood peak occurs. However, few studies have noted that the survival of fish exposed to high-level TDG supersaturation (more than 120%) after experiencing chronic exposure. Bighead carp is an important commercial pelagic fish, dwelling in the Yangtze River. In comparison to the benthic species of China, this species is more susceptible to TDG because the solubility of dissolved gas increases by approximately 10% in conjunction with 1 m of depth increases [22]. Moreover, the juvenile bighead carp in the filed usually migrate from the middle and lower reaches of the Yangtze River to the upper reaches (main nursery ground) from June to October. The juvenile bighead carp are vulnerable to TDG supersaturated water during the migration [23]. In recent years, populations of bighead carp have decreased due to the destruction of habitat since hydropower projects have been put into operation [24,25]. The goal of this study was to evaluate the tolerance of bighead carp to TDG supersaturation and determine the effect of a high level of TDG supersaturation on its survival after chronic TDG exposure. The data from this study may provide valuable information related to protecting aquatic organism diversity and establishing water quality standards related to TDG supersaturation.

## 2. Materials and Methods

### 2.1. Experimental Fish

The bighead carp is one of the four traditional commercial fishes (along with grass carp, silver carp, and black carp) in China and has high economic value. This fish is a dominant natural germplasm resource in the Yangtze River [26] and is naturally distributed in the freshwater area of China. Fish used in this study were obtained from Meishan Tianhe Fisheries Company limited (Meishan, Sichuan, China).

### 2.2. Experimental Devices 

TDG supersaturated water was generated by a system that is described in detail by Li et al., (2019) [27]. In the system, a compressor produced compressed air, which was injected into an autoclave in addition to the water from the water flume to create high TDG supersaturated water. The water was mixed with equilibrium water to obtain varied levels of TDG supersaturated water by controlling their flows. The TDG value rose gradually and achieved the desired TDG level. Throughout the trial, the TDG level and temperature were detected by utilizing a Point 4 Tracker (Point Four Systems, Coquitlam, BC, Canada). A heater was used to maintain the water temperature. A pH metre and a dissolved oxygen (DO) metre were employed to measure the pH and DO level, respectively. An electronic balance and ruler were employed to measure the weight and fork length of the dead fish, respectively.

### 2.3. Acute Lethality Experiment 

In the experiment, two different sized bighead carps were chosen to investigate the effect of TDG on survival. The weight and fork length of the large juvenile bighead carp were 1.00–1.36 g and 4.5–6.1 cm, respectively. The weight and fork length of the small juvenile bighead carp were 0.11–0.27 g and 2.0–2.8 cm, respectively.

Based on the existing survey, the levels of TDG supersaturation were primarily distributed within 120–140% in the lower reaches of most high dams in the flood season [4,28]. TDG supersaturation induced by the flood discharge can maintain a high level over a long distance (180 km) in the lower reach [29]. In this trial, five TDG supersaturation levels (100, 125, 130, 135, and 140%) were set-up for bighead carp. Prior to the experiment, a tank containing 720 L saturated equilibrium water (TDG: 100%, temperature: 22 ± 0.5 ℃ (mean ± SD), DO: 7.2 ± 0.6 mg/L, and pH: 7.6 ± 0.2) was used to temporarily rear experimental fish for 96 h so that they could acclimate to a new water environment. Bighead carp stopped feeding for 24 h before starting the experiment to avoid the influence of feeding. After acclimation, 100 healthy fish were transferred to five tanks (height: 0.5 m, length: 0.5 m, width: 0.5 m, water depth: 0.4 m, 145 L water, and 20 fish per tank) with five TDG supersaturation levels (100, 125, 130, 135, and 140%). The control group was 100% TDG. Three replicates were set up for each group. During the experiment, the water quality condition was consistent with the acclimation. TDG was measured every 1 h. GBD symptoms and abnormal behaviours (e.g., breathing rapidly, swimming up and down at a fast speed, and exhibiting an agitated escape response) were monitored every 5 min through manual observation and recorded. In the experiment, if the test fish stopped swimming and breathing, drifting on the water surface without any reaction to multiple touches, it can be concluded that the test fish was dead. For each dead bighead carp, the time of death, fork length and body weight were recorded.

### 2.4. Acute Lethality Experiment After Chronic TDG Exposure

Generally, TDG saturation varies with flood discharge flow, and high TDG levels regularly result from high levels of flood discharge. Bighead carp may experience long-term chronic exposure with a low level of TDG before the arrival of the flood peak. In this experiment, large juvenile bighead carp with similar size to those used in the above acute experiment were selected to explore the effect of TDG on survival after experiencing chronic TDG exposure. The weight and fork length of the test fish were 1.12–1.68 g and 4.5–6.8 cm, respectively.

Before conducting the chronic exposure test, bighead carp were resettled in a 720 L tank containing saturated equilibrium water (TDG: 100%, temperature: 22 ± 0.7 ℃, DO: 7.5 ± 0.4 mg/L, and pH: 7.3 ± 0.3) to adapt the new water environment. Bighead carp feeding was stopped for 24 h before the experiment began. After acclimation, 500 large juvenile bighead carp were moved to a 720 L experimental tank and exposed to TDG supersaturated water (115%) for 96 h. During chronic exposure, the water quality parameters were consistent with the acclimation conditions. GBD symptoms, abnormal behaviours, and mortality of bighead carp were recorded. After chronic exposure, 300 live fish were selected for the acute exposure experiment. In this acute TDG exposure trial, five TDG levels equal to those in the above acute exposure trials (100, 125, 130, 135, and 140%) were set-up. The juveniles were equally divided into five tanks (20 fish per tank) with five TDG supersaturation levels (100, 125, 130, 135, and 140%). The control group was 100% TDG. Three replicates were set up for each group. During the experiment, the water quality conditions were consistent with the acclimation conditions. TDG was measured every 1 h. GBD symptoms and abnormal behaviours (e.g., breathing rapidly, swimming up and down at a fast speed, and exhibiting an agitated escape response) of the test fish were observed and recorded every 5 min throughout the whole experiment. Parameters for each dead fish including the time of death, fork length and body weight were also recorded.

### 2.5. Statistical Analysis

Differences in fork length and weight of the two sizes of bighead carp were analysed in R version 3.6.1, followed by a generalized two-sample Wilcoxon test. The results were defined as statistically significant if *p* < 0.05. 

To investigate the effect of TDG supersaturation on the survival of bighead carp, the survival probability was utilized to assess the survival process of bighead carp subjected to the TDG supersaturated water. The calculation formula of survival probability is described as follows:

P=(1−nN)∗100%

where P is the survival probability of bighead carp, n is the number of dead fish in each case and N is the total number of the test fish.

Furthermore, the accelerated failure time (AFT) model was applied in the data processing [30]. For the data analysis, a whole distribution of time-to-event (death) was utilized to differentiate the impact degree of the different levels of TDG [31]. The AFT model used here can be described as follows:

$$T=\emptyset \left(Z\right)*T_{0},$$

where T is the time-to-death of bighead carp in the exposure groups, and $T_{0}$ is the time-to-death of bighead carp in the control group. ∅(Z) is a function related to TDG level of the exposure, which is described as follows:

∅(Z)=exp(βd),

where d is a variable representing the TDG level set up for bighead carp, and β is an unknown coefficient that represents the effect of TDG on the time-to-event (death) of exposed bighead carp. Hypothetically, $T_{0}$ followed a loglogistic distribution, and the TDG levels were categorical variables for fitting the survival data. The parameters involved in the AFT model fitting were estimated utilizing the maximum likelihood method. According to the above information, the survival S(t) at a time point (t) in the AFT model was calculated with the following equation:

S(t)=(1+(t·exp(μ)−1·exp(βT)−1)1σ)−1,

where μ (intercept) and σ (scale) are both unknown parameters that can be estimated from the AFT model. The survival curve exhibits the survival process of the study subjects [32]. The descent rate of the survival curve was demonstrated clearly through a stiff (or flattened) curve. The median survival time (ST_50_) was a time point that possessed a 50% survival probability [33]. The ST50 computed via the survfit function was considered to evaluate the tolerance of bighead carp to TDG supersaturated water. The significant differences in the survival curves among the tested groups and the control group were analysed by the survdiff function [30,34]. The fitting of the AFT model and the plotting of the survival curves were performed utilizing the survival, eha and ggplot2 packages in R version 3.6.1 (https://www.r-project.org/).

## 3. Results

**Figure 1:**
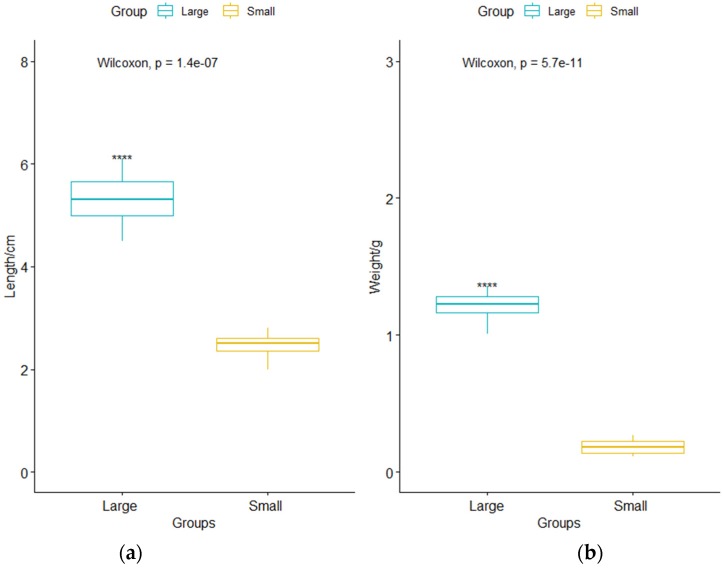
Boxplot describes the difference in the weight and fork length between the large juvenile bighead carp and small juvenile bighead carp. (**a**) The difference in the fork length of the two sizes of bighead carp. (**b**) The difference in the weights of the two sizes of bighead carp. Asterisks indicate that large juvenile bighead carp are significantly different from the small juvenile bighead carp (****: *p* < 0.01).

There was a statistical distinction in the weight and fork length between the two sizes of bighead carp (Wilcoxon test: n = 120, *p* < 0.01) (Figure 1). The weight and fork length of the large juvenile bighead carp were 1.22 ± 0.09 g and 5.27 ± 0.38 cm (mean ± SD), respectively. The weight and fork length of the small juvenile bighead carp were 0.18 ± 0.05 g and 2.50 ± 0.21 cm, respectively.

### 3.1. Symptoms of GBD 

**Figure 2:**
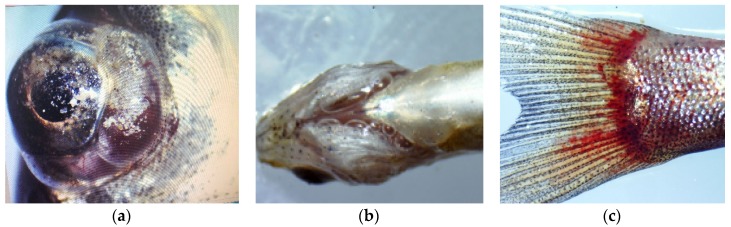
Typical symptoms of gas bubble disease (GBD), (**a**) exophthalmos, (**b**) bubbles in the gills and (**c**) haemorrhage on caudal fin.

When the experiment initially began, fish swam actively in all levels of TDG supersaturated water. Twenty minutes later, some small viscous bubbles were found on the skin of the bighead carp and the surface of the water (140% TDG). Subsequently, the same phenomenon was also observed at other TDG levels (135, 130 and 125%). Furthermore, after 1 h of exposure, the fish in TDG supersaturated water with high levels (140, 135, and 130%) exhibited a series of abnormal swimming behaviours (e.g., sidestroke with rapid breath, agitated escape response and spiral swimming at a fast speed). With an increased duration of exposure (1.5 h), some test fish began to lose their swimming ability (the test fish lost the equilibrium, become disoriented and remained motionless on the water surface). After 2 h, the test fish died and drifted on the surface of the water in the 140% TDG supersaturated water. Similar phenomena were also observed in the bighead carp subjected to 125% TDG supersaturated water. The typical GBD symptoms observed in the dead fish included exophthalmos, bubbles in the gills, and haemorrhages on the fins (Figure 2). 

### 3.2. Acute Effects of TDG on the Survival of Bighead Carp

**Figure 3:**
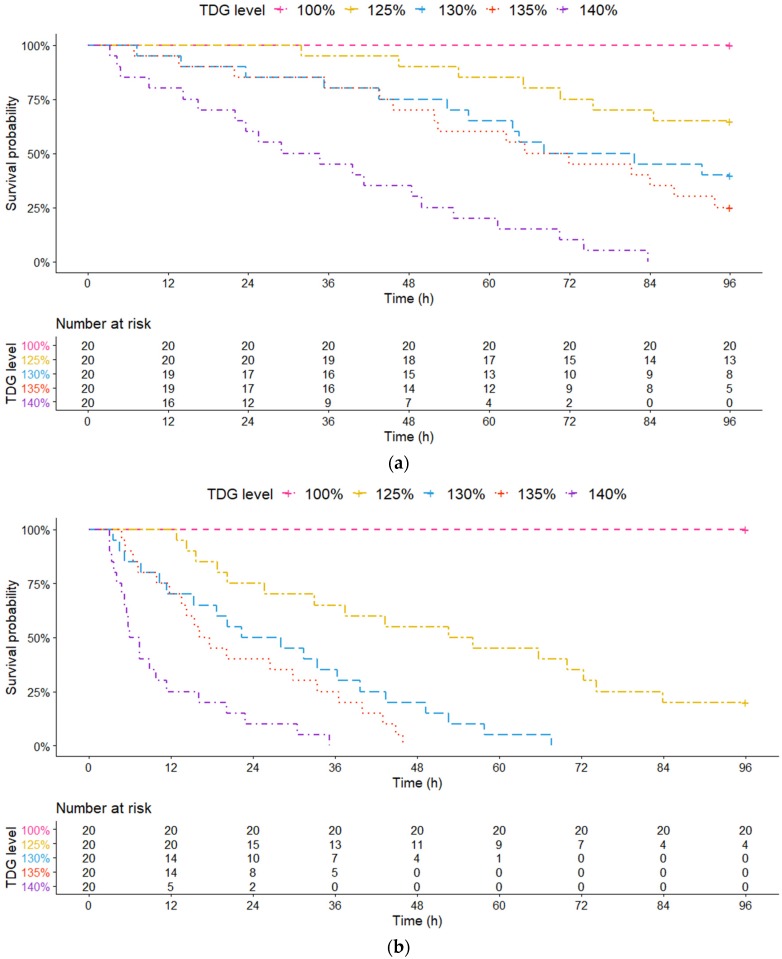
Survival curves of the large and small juvenile bighead carp exposed to five different levels of total dissolved gas (TDG) supersaturated water (100, 125, 130, 135, and 140%). (**a**) Large juvenile bighead carp; (**b**) Small juvenile bighead carp. Broken lines indicate the correlation of survival probability and exposure time. The risk table describes the number of test fish threatened by TDG at different exposure times.

The survival probability declined with increasing TDG level in both large and small juvenile bighead carp. At the 140% TDG level, the large juvenile bighead carp reached a 0% survival probability within 84 h (Figure 3a). However, the survival times (0% survival probability) were approximately 35, 46 and 68 h in the small samples at 140%, 135% and 130% TDG, respectively (Figure 3b). At the 135 and 130% TDG level, the survival probabilities of 25% and 40% of the large fish were obtained from 93 to 96 h and 91 to 96 h, respectively. Furthermore, a high survival probability in the large juvenile bighead carp was found at 125% TDG level until the experiment ended. The survival probability remained 65% for the large juvenile bighead carp, while it was only 20% for small juvenile bighead carp. Moreover, no test fish died at the 100% TDG level in both large and small groups at the end of the experiment. 

The risk table below the survival curve describes the number of test fish exposed to TDG supersaturated water at the risk of death at various exposure times (Figure 3). After 60 h of exposure, the number of large juvenile bighead carp that were at high risk of death were 17, 13, 12 and 4 at the 125, 130, 135, and 140% TDG level, respectively; 9 and 1 small juvenile bighead carp were threatened at 125 and 130%TDG level, respectively; no fish survived at the 135 and 140% TDG levels. 

The output parameters involved in the AFT model fitting are listed in Table 1. TDG significantly influenced the survival of large and small juvenile bighead carp (*p* < 0.01). According to the AFT model, among the TDG levels, the 140% TDG level resulted in the greatest threat to the survival of small and large juvenile bighead carp than any other TDG levels (small juvenile bighead carp: |Coeff $\beta_{130\%}$| = 0.77, *p* < 0.01, |Coeff $\beta_{135\%}$| = 0.99, *p* < 0.01, and |Coeff $\beta_{140\%}$| = 1.87, *p* < 0.01; large juvenile bighead carp: |Coeff $\beta_{130\%}$| = 0.54, *p* = 0.08, |Coeff $\beta_{135\%}$| = 0.70, *p* = 0.02, and |Coeff $\beta_{140\%}$| = 1.50, *p* < 0.01).

The median survival time (ST_50_) of bighead carp was calculated from the relevant survival curve. TDG had a significant influence on the change in the ST_50_ (Table 2). Both large and small juvenile bighead carp experienced a decrease in ST_50_ with increasing TDG level. The ST_50_ of the large and small juvenile bighead carp was 74.97 and 22.40 h, respectively, at 130% TDG. The ST_50_ decreased by over half at 140% TDG (31.90 and 6.72 h). Compared with the ST_50_ of the small fish (22.40, 16.93 and 6.72 h), that of the large fish increased (74.97, 68.60 and 31.90 h) at all the TDG levels and exhibited a greater tolerance to TDG supersaturation. 

### 3.3. Acute Effects of TDG on the Survival of Bighead Carp after Chronic Exposure

No fish died throughout the chronic exposure trial, and a small number of test fish exhibited slight GBD symptoms. No abnormal behaviour was observed in the test fish. Furthermore, bighead carp subjected to acute re-exposure exhibited similar GBD symptoms to those in the above acute exposure. All fish survived at the 100% TDG level at the end of the experiment in the acute re-exposure trial. 

**Figure 4:**
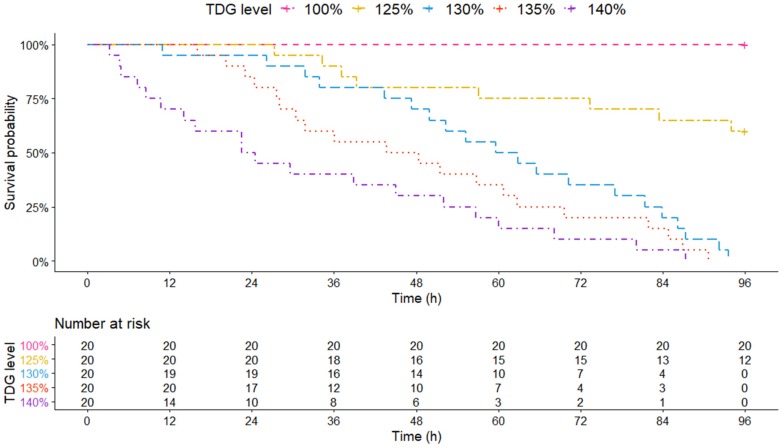
Survival curves of large juvenile bighead carp exposed to five different levels of TDG supersaturated water (100, 125, 130, 135, and 140%) after chronic exposure (115%). Broken lines indicate the correlation of survival probability and exposure time. The risk table illustrates the number of test fish threatened by TDG at various exposure times.

TDG resulted in increased stress on bighead carp survival as the TDG levels increased. At approximately 9, 28 and 43 h, the survival probability of bighead carp decreased to 75% at the 140, 135, and 130% TDG levels, respectively (Figure 4); at the same levels, the survival probability of bighead carp reached 0% at approximately 87, 91, and 94 h, respectively. Bighead carp maintained a 60% survival probability at 125% TDG at the end of the experiment. No fish died at the 100% TDG level until the experiment ended.

The risk table below the survival curve illustrates the quantity of bighead carp exposed to TDG supersaturated water at the risk of death at various exposure times (Figure 4). At 48 h, the number of test fish at the risk of death from TDG were 16, 14, 10, and 6 (at 125, 130, 135, and 140%, respectively). At 96 h, 12 fish survived at 125% TDG, while no fish survived at 130, 135, and 140% TDG.

The results of parameter estimation associated with the AFT model fitting are listed in Table 3. TDG significantly impacted the survival of bighead carp (*p* < 0.01). Compared with 130 and 135% TDG, the effect of 140% TDG on the survival of bighead carp was greater in this trial (|Coeff $\beta_{130\%}$| = 0.64, *p* < 0.01, |Coeff $\beta_{135\%}$| = 0.92, *p* < 0.01, and |Coeff $\beta_{140\%}$| = 1.52, *p* < 0.01).

The ST_50_ of bighead carp subjected to acute stress after chronic exposure is shown in Table 4. With increasing TDG supersaturation levels, the ST_50_ of bighead carp declined rapidly. At 130% TDG, the ST_50_ of the test fish was 61.23 h, which was almost three times of that at 140% TDG (23.50 h).

## 4. Discussion

Previous studies have illustrated that increasing TDG levels causes adverse effects on the survival of fish due to GBD [15,18,35]. TDG supersaturation led to the formation of emphysema or embolism in the gill blood vessels, which obstructed blood circulation and disrupted the normal operation of the respiratory system of the fish, resulting in the death of the fish due to anoxia [36,37]. The symptoms of GBD, including exophthalmos, haemorrhages in the fins, swelling of the swim bladder, and emboli in blood vessels, have been demonstrated in the previous studies [35,38,39]. In the present study, the GBD symptoms in bighead carp were consistent with the above-described symptoms. Furthermore, abnormal behaviours of fish subjected to TDG were also observed. Waytt and Beiningen (1971) found that fish suffering from acute exposure lose their lateral line function in addition to swimming ability, which resulted in a lack of capacity to avoid predators and obstacles. In addition, some actions, such as sidestroking accompanied by rapid breath, swimming up and down rapidly and exhibiting an agitated escape response, were also indicated in previous studies [40,41]. Analogous abnormal activities in bighead carp were observed in this study. 

It has been suggested that fish size might play an important role in fish tolerance to TDG. Dawley et al., (1976) [42] indicated the survival probability of several sizes salmon subjected to supersaturated water with 112% TGP (total gas pressure), small salmon (40 mm long) remained high (>90%) over 45 days, while large fish (53 mm and 67 mm long) maintained a lower survival probability (<50%) over only half a month. Furthermore, Rucker (1975) [43] demonstrated that the median lethal time (LT50, 50% survival probability) of large coho salmon was almost one-tenth that of small fish at 112% TGP supersaturated water. These studies indicated that the tolerance of juvenile salmonids to TDG supersaturation decreased with increasing size. In this study, both large and small juvenile bighead carp showed a decline in survival probability as the TDG levels increased. This indicated that increasing TDG levels greatly influenced the survival of bighead carp. By comparing the survival curves of two sizes of juvenile bighead carp, the large juvenile bighead carp reached a 0% survival probability within 84 h at the 140% TDG level while all small juvenile survived approximately for 35 h at the same TDG level (Figure 3a,b). Moreover, a high survival probability (65%) in the large juvenile bighead carp was found at the 125% TDG level at the end of the experiment. However, the small juveniles only had 20% survival probability. In comparison to the large juvenile bighead carp, the small juvenile bighead carp were more sensitive to TDG. Furthermore, the ST_50_ (50% survival probability) values of the large fish were 74.97, 68.60 and 31.90 h at 130, 135, and 140% TDG, respectively, while the ST_50_ values of the small fish were 22.40, 16.93 and 6.72 h at the same TDG levels. In comparison to the small juvenile bighead carp, the large juvenile bighead carp possessed a longer ST_50_ at all TDG levels. The tolerance of bighead carp was enhanced with increasing size. In addition, these data (ST_50_) can be treated as valuable information for formulating the scheme of reservoir operation in the Yangtze River basin. In China, previous studies have shown that different benthic endemic species in the Yangtze River have diverse tolerance to TDG. According to Li et al., (2019), the LT_50_ (50% survival probability) values of the Chinese sucker were 6.1, 4.5 and 3.1 h at 130, 135, and 140% TDG, respectively. In addition, Wang et al., (2015) [39] indicated that Prenant’s schizothoracin in TDG supersaturated water at levels of 130, 135, and 140% possessed LT_50_ values of 8.7, 8.0 and 4.6 h, respectively. Huang et al., (2010) [42] noted that the LT_50_ values of rock carp were 8.2, 6.6 and 3.5 h, respectively. Compared with these fishes, the large juvenile bighead carp showed a higher tolerance to TDG supersaturation. 

Furthermore, according to Liu et al., (2011) [19], rock carp were subjected to chronic TDG exposure (at 104, 108, 112 and 116%). Their results showed that chronic exposure to TDG induced oxidative stress in rock carp and led to cell injury. Yuan et al., (2017) [44] suggested that *Leptobotia elongata* subjected to chronic exposure of TDG (110 and 120%) for 96 h showed weak tolerance during subsequent acute exposure (134 and 140%). In this study, at 96 h postexposure to TDG supersaturated water at a level of 115%, no bighead carp death was observed. In the subsequent acute re-exposure, all juveniles survived at the 100% TDG level. The results of this study also indicated that the ST_50_ values of bighead carp subjected to multiple exposures (acute exposure after chronic exposure) were 61.23, 55.50 and 23.50 h at 130, 135, and 140%, respectively, however, at the same TDG levels, the ST_50_ values of bighead carp that experienced only acute exposure were 74.97, 68.60 and 31.90 h, respectively. Compared with the acute exposure group, fish subjected to multiple exposures had lower ST_50_ values and were more susceptible to multiple TDG exposures. Bighead carp have likely been injured from chronic exposure before acute exposure [19]. Furthermore, on the basis of the output results of the AFT model fitting (Table 1 and Table 3), TDG significantly impacted the survival of bighead carp in this study (multiple exposures: |Coeff $\beta_{130\%}$| = 0.64, *p* < 0.01, |Coeff $\beta_{135\%}$| = 0.92, *p* < 0.01, and |Coeff $\beta_{140\%}$| = 1.52, *p* < 0.01; single acute exposure: (large juvenile bighead carp) |Coeff $\beta_{130\%}$| = 0.54, *p* = 0.08, |Coeff $\beta_{135\%}$| = 0.70, *p* = 0.02, and |Coeff $\beta_{140\%}$| = 1.50, *p* < 0.01; (small juvenile bighead carp) |Coeff $\beta_{130\%}$| = 0.77, *p* < 0.01, |Coeff $\beta_{135\%}$| = 0.99, *p* < 0.01, and |Coeff $\beta_{140\%}$| = 1.87, *p* < 0.01).

Previous studies have stated that the AFT model can be utilized to estimate and quantify the impact of toxicants on the survival of organisms [25,26,29]. As our study shows, the pattern of the recorded time-to-death data from the TDG exposure experiment can feasibly be explained by the AFT model. At the same time, the estimated parameters applied to evaluate the statistical importance and describe the effect of TDG can also be obtained through the AFT model. In the process of model fitting, the exposure time and TDG can be integrated into a survival analysis. The risk evaluation efficiency involved with TDG was promoted by applying the AFT model [29]. Through the comparison of the absolute value of the parameters, we could intuitively conclude which level of TDG had the most significant influence on bighead carp survival.

## 5. Conclusions

Overall, our study indicates that high TDG levels in the supersaturated water cause a great threat to the survival of bighead carp. In comparison to the large juvenile bighead carp, the small juvenile bighead carp show lower tolerance to TDG and are more sensitive to TDG. The 115% TDG did not cause the bighead carp death, and 20–65% of bighead carp still survived at 125% for 96 h. We suggest that 120% TDG should be treated as a tolerance threshold for this species. Furthermore, bighead carp subjected to acute exposure after 96 h chronic exposure are more vulnerable to TDG, and some effective measures should be proposed to protect the species from TDG supersaturation in China. In our study, the results also suggest that the AFT model can efficiently evaluate the influence of TDG on fish survival. 

## Figures and Tables

**Table 1:** Regression coefficients for the accelerated failure time (AFT) model depicting the effects of TDG on the survival (time-to-death) of two different sizes of bighead carp.

Groups	Factors	Parameters	Estimated Coefficients (Coeff.)		
			Coeff. (S.E.) ^1^	Walid-Z	Pr(>|Z|) ^2^
Large	Intercept	μ	4.90 (0.24)	20.73	<0.01 **
	TDG	β	−0.54 (0.31)_130%_	−1.75	0.08
			−0.70 (0.31)_135%_	−2.31	0.02 *
			−1.50 (0.31)_140%_	−4.93	<0.01 **
	Log (scale)	σ	−0.68 (0.12)	−5.73	<0.01 **
					
Small	Intercept	μ	3.89 (0.19)	20.69	<0.01 **
	TDG	β	−0.77 (0.27)_130%_	−2.91	<0.01 **
			−0.99 (0.26)_135%_	−3.80	<0.01 **
			−1.87 (0.26)_140%_	−7.19	<0.01 **
	Log (scale)	σ	−0.76 (0.09)	−8.09	<0.01 **

**^1^** TDG is considered a categorical variable for the fitting survival data. **^2^** Asterisks represent significance (*: *p* < 0.05; **: *p* < 0.01); S.E.: standard error.

**Table 2:** Acute effect of TDG supersaturation on the median survival time (ST_50_), and statistical importance of the survival curves of two different sizes of bighead carp.

Groups	TDG Level	ST_50_ (h)	Statistical Importance		
			df	Chi-square	Pr(>Chi-sq) ^1^
Large	125%				
	130%	74.97 ± 0.81	1	3.0	0.08
	135%	68.60 ± 0.61	1	6.6	0.01 *
	140%	31.90 ± 1.31	1	30.7	<0.01 **
Small	125%	54.36 ± 4.16			
	130%	22.40 ± 3.19	1	10.8	<0.01 **
	135%	16.93 ± 1.65	1	16.0	<0.01 **
	140%	6.72 ± 1.46	1	30.0	<0.01 **

**^1^** Asterisks represent significance (*: *p* < 0.05; **: *p* < 0.01).

**Table 3:** Regression coefficients for the accelerated failure time (AFT) model depicting the effects of TDG on the survival (time-to-death) of bighead carp.

Factors	Parameters	Estimated Coefficients (Coeff.)		
		Coeff. (S.E.) ^1^	Walid-Z	Pr(>|Z|) ^2^
Intercept	μ	4.69 (0.19)	25.22	<0.01 **
TDG	β	−0.64 (0.24)_130%_	−2.71	<0.01 **
		−0.92 (0.24)_135%_	−3.83	<0.01 **
		−1.52 (0.27)_140%_	−5.75	<0.01 **
Log (scale)	σ	−0.89 (0.10)	−8.74	<0.01 **

**^1^** TDG is considered a categorical variable for fitting the survival data. ^**2**^ Asterisks represent significance (*: *p* < 0.05; **: *p* < 0.01); S.E.: standard error.

**Table 4:** Acute effect of TDG supersaturation after chronic exposure on the median survival time, and statistical importance of the survival curves of bighead carp.

TDG Level	ST_50_ (h)	Statistical Importance		
		df	Chi-square	Pr(>Chi-sq) ^1^
125%				
130%	61.23 ± 1.53	1	16	<0.01 **
135%	55.50 ± 3.20	1	19.9	<0.01 **
140%	23.50 ± 1.25	1	25.7	<0.01 **

^1^ Asterisks represent significance (**: *p* < 0.01).
